# Production of VEGF and expression of the VEGF receptors Flt-1 and KDR in primary cultures of epithelial and stromal cells derived from breast tumours

**DOI:** 10.1038/sj.bjc.6690438

**Published:** 1999-05

**Authors:** V Speirs, S L Atkin

**Affiliations:** Department of Medicine, Medical Research Laboratory, University of Hull, Hull, HU6 7RX, UK

**Keywords:** breast, cancer, VEGF, angiogenesis

## Abstract

Production of vascular endothelial growth factor (VEGF) and expression of its receptors Flt-1 and KDR was determined in primary cultures of separated epithelial and stromal-enriched cultures derived from ten primary human breast carcinomas. By enzyme-linked immunosorbent assay, epithelial cells produced a mean VEGF of 33 ± 7 pg ml^−1^ μg^−1^ RNA (range 11–70). Stromal cells produced similar levels, with a mean of 48 ± 11 pg ml^−1^ μg^−1^ RNA (range 7–92). This was significantly greater than the amount produced by similar cultures derived from normal breast tissue (epithelial mean 19 ± 5 pg ml^−1^ μg^−1^ RNA, range 9–34, *P* < 0.05 vs tumour epithelial culture; stromal mean 26 ± 8 pg ml^−1^ μg^−1^ RNA, range 3–56). Flt-1 and KDR receptors were analysed by semi-quantitative reverse transcription polymerase chain reaction. Flt-1 was expressed by four of six epithelial and five of six stromal cultures. When expressed by both cell types, Flt-1 appeared to be significantly more abundant on stromal cells compared with epithelial cultures. Only a single tumour, a lobular carcinoma, failed to express Flt-1 on either cell type. With KDR, the reverse was true with constitutive expression of this receptor by epithelial cultures and zero or reduced (3/6) expression by stromal cultures. Differences in the expression pattern of VEGF receptors may reflect a differential response to VEGF by specific cell types. Thus, production of VEGF and expression of VEGF receptors Flt-1 and KDR by breast cancer epithelial and stromal cells suggests that VEGF may fulfil not only an angiogenic role, but also play a fundamental role as an autocrine/paracrine regulator in breast cancer, thereby facilitating tumour proliferation and subsequent invasion. © 1999 Cancer Research Campaign


*British Journal of Cancer* (1999) **80**(5/6), 898–903
© 1999 Cancer Research Campaign
Article no. bjoc.1998.0438

### Production of VEGF and expression of the VEGF receptors Flt-1 and KDR in primary cultures of epithelial and stromal cells derived from breast tumours

**V Speirs and SL Atkin**

Department of Medicine, Medical Research Laboratory, Wolfson Building, University of Hull, Hull HU6 7RX, UK

**Summary** Production of vascular endothelial growth factor (VEGF) and expression of its receptors Flt-1 and KDR was determined in primary cultures of separated epithelial and stromal-enriched cultures derived from ten primary human breast carcinomas. By enzyme-linked immunosorbent assay, epithelial cells produced a mean VEGF of 33 ± 7 pg $ml^{-1}$ $\mu g^{-1}$ RNA (range 11–70). Stromal cells produced similar levels, with a mean of 48 ± 11 pg $ml^{-1}$ $\mu g^{-1}$ RNA (range 7–92). This was significantly greater than the amount produced by similar cultures derived from normal breast tissue (epithelial mean 19 ± 5 pg $ml^{-1}$ $\mu g^{-1}$ RNA, range 9–34, $P < 0.05$ vs tumour epithelial culture; stromal mean 26 ± 8 pg $ml^{-1}$ $\mu g^{-1}$ RNA, range 3–56). Flt-1 and KDR receptors were analysed by semi-quantitative reverse transcription polymerase chain reaction. Flt-1 was expressed by four of six epithelial and five of six stromal cultures. When expressed by both cell types, Flt-1 appeared to be significantly more abundant on stromal cells compared with epithelial cultures. Only a single tumour, a lobular carcinoma, failed to express Flt-1 on either cell type. With KDR, the reverse was true with constitutive expression of this receptor by epithelial cultures and zero or reduced (3/6) expression by stromal cultures. Differences in the expression pattern of VEGF receptors may reflect a differential response to VEGF by specific cell types. Thus, production of VEGF and expression of VEGF receptors Flt-1 and KDR by breast cancer epithelial and stromal cells suggests that VEGF may fulfil not only an angiogenic role, but also play a fundamental role as an autocrine/paracrine regulator in breast cancer, thereby facilitating tumour proliferation and subsequent invasion.

**Keywords:** breast; cancer; VEGF; angiogenesis

Angiogenesis is critical for growth and metastatic spread of solid tumours (Folkman, 1990) and is mediated by a number of angiogenic growth factors, of which vascular endothelial growth factor (VEGF) is the most potent inducer neovasculature (reviewed in Klagsbrun and Soker, 1993). The VEGF gene consists of eight exons that give rise to four different isoforms of VEGF (121, 165, 189 and 206 amino acids) by alternative splicing (Tischer et al, 1991). In both normal and transformed cells, $VEGF_{165}$ appears to be the most abundant gene product (Ferrara and Davis-Smyth, 1997). VEGF binds to specific high-affinity tyrosine kinase receptors, termed Flt-1 and KDR, which mediate the VEGF response (de Vries et al, 1992; Terman et al, 1992). Both receptors are closely related and display 44% sequence homology, although Flt-1 has highest affinity for recombinant human VEGF (rhVEGF) (de Vries et al, 1992).

VEGF is produced by a number of tumours, including that of the breast, and compared with other angiogenic factors, e.g. basic fibroblast growth factor (bFGF), transforming growth factor (TGF)-$\alpha$ and TGF-$\beta$, its production by breast tumours is significantly up-regulated compared with adjacent normal tissue (Yoshiji et al, 1996). The importance of VEGF-induced neovascularization in breast tumours may be seen from recent clinical studies which have shown that the degree of tumour angiogenesis is an important prognostic factor (Weidner et al, 1991; Toi et al, 1993). Further, VEGF is one of the most important factors in promoting neovascularization of breast tumours (Toi et al, 1996). The clinical prognostic value of VEGF has been substantiated by two recent studies that demonstrate that the level of $VEGF_{165}$ is a strong, independent prognostic determinant (Eppenberger et al, 1998; Linderholm et al, 1998).

Although VEGF is clearly an important factor in the angiogenic process in breast cancer, there is scant information about the production of bioactive VEGF or expression of VEGF receptors by specific cell types in the breast. Therefore, the first aim of this study was to determine the production of VEGF by primary cultures of breast epithelial and stromal-enriched cells derived from human breast tumours and its modulation by the cytokines interleukin (IL)-6 and IL-8. These factors were selected as they have both previously been shown to be up-regulated in breast cancer (Adams et al, 1991; Green et al, 1997) and both induce angiogenesis (Koch et al, 1992; Cohen et al, 1996). Secondly, we determined the in vitro expression of the VEGF receptors, Flt-1 and KDR and assessed whether they may mediate autocrine/paracrine proliferation.

### METHODS

### Cell culture

Surgically removed breast tumours ($n$ = 10) were transported immediately to the laboratory and dispersed overnight in collagenase III (Life Technologies, Paisley, UK) as previously described (Speirs et al, 1996*a*). Individual epithelial and stromal preparations were isolated using a differential centrifugation method followed by culture in selective media (Speirs et al, 1996*b*) and were previously fully characterized by immunostaining, flow

*Received 26 August 1998*
*Revised 30 November 1998*
*Accepted 9 December 1998*

*Correspondence to:* V Speirs

**Table 1** Production of VEGF and expression of VEGF receptors KDR and Flt-1 in cultures of primary epithelial and stromal cells derived from breast tumours

| Sample | | VEGF (pg ml$^{-1}$ μg$^{-1}$ RNA) | GAPdH | KDR | Flt-1 | Age | Type | Grade | LN | ER |
|---|---|---|---|---|---|---|---|---|---|---|
| BCa1 | E | 13 | NA | NA | NA | 63 | Ductal | II | – | + |
| | S | 39 | NA | NA | NA | | | | | |
| BCa2 | E | 38 | + | + | – | 56 | Lobular | I | – | + |
| | S | 7 | + | + | – | | | | | |
| BCa3 | E | 70 | NA | NA | NA | 80 | Ductal | II | – | – |
| | S | 59 | NA | NA | NA | | | | | |
| BCa4 | E | 27 | + | + | + | 56 | Ductal | II | + | – |
| | S | 75 | + | + | + | | | | | |
| BCa5 | E | 11 | NA | NA | NA | 72 | Ductal | II | + | + |
| | S | 35 | NA | NA | NA | | | | | |
| BCa6 | E | 51 | NA | NA | NA | 68 | Ductal | II | + | + |
| | S | 67 | NA | NA | NA | | | | | |
| BCa7 | E | 36 | + | + | + | 56 | Ductal | III | – | – |
| | S | 92 | + | + | + | | | | | |
| BCa8 | E | 21 | + | + | + | 72 | Ductal | II | + | + |
| | S | 7 | + | – | + | | | | | |
| BCa9 | E | NA | + | + | + | 55 | Ductal | II | – | – |
| | S | NA | + | – | + | | | | | |
| BCa10 | E | NA | + | + | – | 47 | Ductal | I | – | – |
| | S | NA | + | – | + | | | | | |

LN, lymph node; ER, oestrogen receptor; BCa, breast cancer; E, epithelial culture; S, stromal culture; NA = not analysed.

cytometry, gene expression and enzyme assay (Speirs et al, 1998). Clinicopathological details of the tumours from which the cultures were established are presented in Table 1. Six normal breast samples from patients undergoing reduction mammoplasty were dispersed enzymatically and cultured under the same conditions as the tumour tissue. Ethical permission was granted for all the studies performed.

### RNA extraction and cDNA synthesis

Total RNA was extracted from pre-confluent cell cultures by the addition of guanidinium isothiocyanate directly to the culture vessel, ethanol precipitated and quantified by UV spectroscopy. This was reverse transcribed into cDNA in a 20-μl reaction containing 1 μg RNA, 2 μg oligo dT, 20 units of RNAsin (both Pharmacia, St Albans, UK), 10 mM dithiothreitol (DTT), 0.1 mM of each deoxynucleotide triphosphate (Boehringer Mannheim, Lewes, UK), 10 units μl$^{-1}$ of SuperScript RNase H$^{-}$ reverse transcriptase (Life Technologies) and an appropriate volume of diethyl pyrocarbonate-treated water. This was incubated for 1 h at 42°C, heated to 99°C for 5 min, quick chilled on ice and stored at –20°C. Polymerase chain reaction (PCR) was performed in a 50-μl volume which contained the following: 2 units of DNA polymerase, 10 × PCR buffer (containing 1.5 mM magnesium chloride; both Bioline, London, UK), 0.5 μg of each oligonucleotide primer (Life Technologies), 200 mM each of dATP, dCTP, dTTP and dGTP, 2 μl nascent cDNA and sterile distilled water to bring the volume to 50 μl.

### Semi-quantitative reverse transcription-PCR

Primer sequences for amplification of Flt-1 and the constitutively expressed housekeeping gene glyceraldehyde 3-phosphate dehydrogenase (GAPDH), have been previously described (de Vries et al, 1992; Green et al, 1996). PCR was performed as follows: 2 μl of cDNA were amplified over 30 cycles with 2 U Bio *Taq* polymerase, 10 × reaction buffer (both Bioline), 200 μM each of dATP, dCTP, dTTP and dGTP (Boehringer Mannheim), 1.5 mM megnesium chloride and distilled water to bring the volume up to 50 μl. For KDR, a nested PCR was performed as originally described (Boocock et al, 1995), but with primer modifications. cDNAs were amplified with KDR-specific primers 1 and 2 for 25 cycles (95°C 30 s, 60°C 30 s, 72°C 30 s). After amplification with the first primer pair, 2 μl of product were reamplified in 50 μl of fresh reaction mixture using the KDR-specific primers 3 and 4 for a further 25 cycles (95°C 30 s, 62°C 30 s, 72°C 30 s). Reaction buffer was identical to that used for Flt-1 and GAPDH. KDR primers were as follows:

Primer 1: 5′-ACG CTG ACA TGT ACG GTC TAT-3′
Primer 2: 5′-TTC CCA TTT GCT GGC ATC ATA-3′
Primer 3: 5′-CAT CAC ATC CAC TGG TAT TGG-3′
Primer 4: 5′-GCC AAG CTT GTA CCA TGT GAG-3′

All PCRs were performed using an OmneGene thermal cycler (Hybaid, London, UK). Substitution of cDNA with either sterile distilled water or non-reverse transcribed RNA served as negative controls; these were consistently negative. A total of 5 μl of each PCR product were electrophoresed through a 2% agarose gel and visualized by ethidium bromide staining under UV illumination. To confirm the identity of the amplified fragments, representative PCR products were incubated with 10% v/v restriction endonuclease (Pvu II for Flt-1, Ava II for KDR; data not shown) which cleaved the product into discrete fragments of predicted size.

### Characterization of cell culture contaminants

To ensure our primary cultures did not contain endothelial contaminants which may have been the source of VEGF/VEGF receptors, all epithelial and stromal cultures were characterized by reverse transcription PCR (RT-PCR) for von Williebraad factor (VWF) which is associated with endothelial cells. RT-PCR was performed as described above using primers designed to detect fragment of the VWF gene. Primer sequences were as follows:

© Cancer Research Campaign 1999

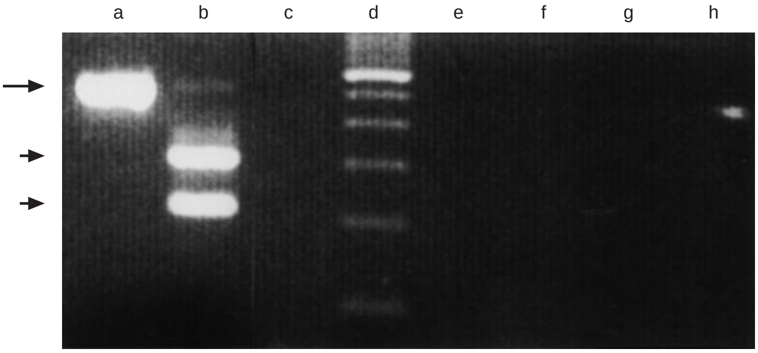

**Figure 1** Detection of VWF in the endothelial cell line EAHY 926 and its absence in four representative primary breast epithelial cultures. Lane a = EAHY 926, lane b = restriction mapped product confirming product identity, lane c = negative control, lane d = 100-bp size standard, lanes e–h = primary breast epithelial cultures. Arrow refers to product size of 509-bp, arrowheads refer to restriction digests of 300 and 209-bp

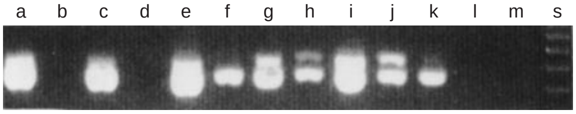

**Figure 2** Detection of Flt-1 receptor in primary epithelial and stromal cultures derived from breast tumours by semi-quantitative RT-PCR. Arrowhead refers to product size of 1080-bp. Lanes a, c, e, g, i, k refer to epithelial cultures and lanes b, d, f, h, j, l are stromal cultures. m = negative control, s = 100-bp size standard

5′-ACG GCT TGC ACC ATT CAG CT-3′
5′-CAG CCT CAC TTG CTG CAC TT-3′

The thermal cycle consisted of a denaturation step of 94°C for 2 min, followed by 35 cycles of 94°C for 30 s, 57.5°C for 30 s, 72°C for 30 s, and was concluded with a final primer extension step of 72°C for 5 min. As a positive control, cDNA obtained from the endothelial cell line EAHY 926 was used. Non-reverse transcribed RNA served as a negative control. Products were analysed by agarose gel electrophoresis as described above.

### Quantitative analysis of VEGF

Primary epithelial and stromal cultures were established in 12-well plates and, when 70% confluent, incubated for 24 h with serum-free medium. The resulting confluent medium (CM) was collected and stored at –80°C until required. From the same cultures, RNA was extracted as described above. CM was quantitatively analysed for VEGF by enzyme-linked immunosorbent assay (ELISA) (R&D Systems, Abingdon, UK). Prior to analysis, CM was centrifuged to remove any particulate matter and assayed according to the manufacturer's instructions. The mean intra- and inter-assay variances were 4.7% and 6.7%, respectively, with a sensitivity of 5 pg $ml^{-1}$ for $VEGF_{165}$. No significant cross-reactivity with a range of cytokines was observed (refer to manufacturer's instructions for complete list).

### Effects of IL-6 and IL-8 on VEGF production by primary epithelial and stromal cultures

Cultures were established from three tumours (BCa2, BCa3 and BCa5) as described above and allowed to become semi-confluent in 12-well plates. Medium was removed and replaced with fresh serum-free medium containing 10 ng $ml^{-1}$ IL-6 or IL-8 (both Genzyme, West Malling, UK). Cultures were incubated with these factors for a further 48 h, after which the medium was collected, clarified by centrifugation and stored at –80°C until analysed for VEGF by ELISA.

### Statistical analysis

Student's unpaired *t*-test was used to analyse statistical significance.

### RESULTS

### Characterization of cell cultures

To ensure the cultures were free from endothelial cell contamination, epithelial and stromal cultures under study were subjected to RT-PCR for the VWF gene. VWF was strongly expressed by the positive control endothelial cell line EAHY 926 and its identity was confirmed by restriction mapping. However, we were unable to detect VWF in our enriched epithelial cell populations (Figure 1) or stromal populations (data not shown). Immunostaining for VWF was also consistently negative (data not shown; Speirs et al, 1996*b*).

### Production of VEGF and expression of VEGF receptors by cell cultures

All breast tumour epithelial and stromal cultures produced VEGF. Epithelial-enriched cultures produced a mean VEGF of 33 ± 7 pg $ml^{-1}$ $\mu g^{-1}$ RNA (range 11–70). Stromal cells produced similar levels, with a mean of 48 ± 11 pg $ml^{-1}$ $\mu g^{-1}$ RNA (range 7–92). This is illustrated in Table 1. For comparison, identical cultures were prepared from six samples of normal breast tissue (obtained from reduction mammoplasty samples). These cultures produced significantly lower amounts of VEGF compared with tumour cultures (epithelial mean 19 ± 5 pg $ml^{-1}$ $\mu g^{-1}$ protein, range 9–34, $P < 0.05$ vs tumour epithelial culture; stromal mean 26 ± 8 pg $ml^{-1}$ $\mu g^{-1}$ protein, range 3–56). We next examined whether the VEGF receptors, Flt-1 and KDR, were expressed by these cultures. The Flt-1 receptor was expressed by four of six epithelial and five of six stromal cultures (Table 1 and Figure 2). Where present in both cell types, under semi-quantitative conditions, Flt-1 appeared to be more abundant on stromal cells compared with epithelial cultures. A single lobular carcinoma, BCa2, failed to express Flt-1 on either cell type. The converse was found with

© Cancer Research Campaign 1999

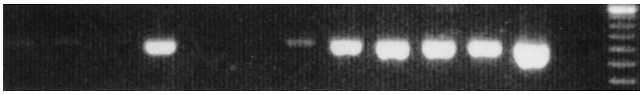

**Figure 3** Detection of KDR receptor in primary epithelial and stromal cultures derived from breast tumours by semi-quantitative RT-PCR. Arrowhead refers to product size of 402-bp. Lanes a, c, e, g, i, k refer to epithelial cultures and lanes b, d, f, h, j, l are stromal cultures. m = negative control, s = 100-bp size standard

KDR with constitutive expression of this receptor by epithelial cultures and zero or reduced (3/6) expression by stromal cultures (Table 1 and Figure 3). To determine the functionality of these receptors, epithelial-enriched cultures were incubated with rh VEGF (5, 10 ng ml$^{-1}$) for up to 48 hours, trypsinized and counted. However, no effects on cell proliferation were observed (data not shown).

### Effect of IL-6 and IL-8 on VEGF production in primary breast cultures

Treatment of epithelial-enriched cultures with either IL-6 or IL-8 had no effect on VEGF production. However, when corresponding stromal cultures were incubated with these factors, VEGF production was decreased by up to 50%. This is illustrated in Figure 4.

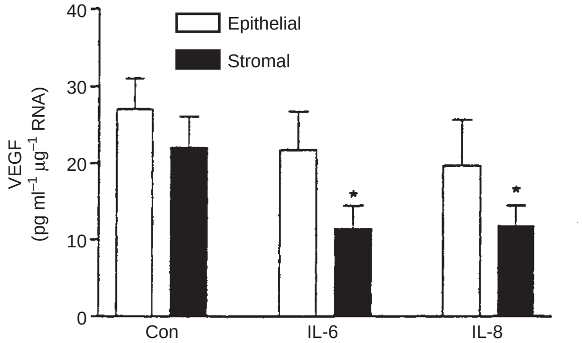

**Figure 4** Effect of IL-6 and IL-8 on production of VEGF by primary breast epithelial and stromal cultures. *$P < 0.05$ vs appropriate epithelial culture

### DISCUSSION

This study has clearly demonstrated production of VEGF and, unexpectedly, expression of the VEGF receptors Flt-1 and KDR by primary cultures derived from breast tumours which were enriched for epithelial and stromal cells. These data suggest that VEGF and its receptors may play a significant autocrine/paracrine role in breast tumorigenesis.

Significantly higher levels of VEGF were produced by tumour epithelial cells compared with normal breast epithelial cells derived from reduction mammoplasties. This is in accordance with the increased expression of VEGF in tumour versus normal breast reported by others (Yoshiji el al, 1996; Scott et al, 1998). This observation suggests that VEGF may be critical for tumour growth and invasion, and is consistent with clinical studies showing that the degree of tumour angiogenesis is an important prognostic factor in breast cancer (Weidner et al, 1991; Toi et al, 1993). Similar production of $VEGF_{165}$ was found for both tumour-derived epithelial and stromal-enriched cultures, suggesting that both cell populations may have equal importance as a source of VEGF. Immunostaining and in situ hybridization studies have revealed that VEGF is more commonly localized to epithelial cells (Brown et al, 1995; Yoshiji et al, 1996). However, it has been shown that mammary stromal cells also produce VEGF which is up-regulated in response to hypoxia (Hlatky et al, 1994), which would be in accord with the results presented here.

To our knowledge this is the first report of expression of Flt-1 and KDR receptors in primary cultures of epithelial and stromal cells derived from breast tumours, although these have previously been noted in established breast cancer cell lines (Lu and Brodie 1996). With the exception of ovarian carcinoma cells (Boocock et al, 1995), melanoma (Gitay-Goran et al, 1993), placental trophoblasts (Charnock-Jones et al, 1994), human testes (Ergun et al, 1997) and monocytes which express Flt-1 but not KDR (Hewett et al, 1996), VEGF receptors are expressed almost exclusively by endothelial cells involved with tumour neovascularization (Jakeman et al, 1992; Hewett and Murray, 1996). Because of the nature of our cell separation prcedure combined with the high sensitivity of the PCR technique, it was possible that we may have been amplifying Flt-1 and KDR message from occult endothelial cells rather than epithelial cells or fibroblasts. However, we have previously characterized these cultures by immunocytochemistry and flow cytometry (Speirs et al, 1998). More importantly, using RT-PCR for the endothelial marker factor VIII, we were unable to detect a positive signal in breast cancer cell cultures, confirming the absence of stray endothelial cells in our epithelial and stromal populations. The sensitivity of the PCR, in theory capable of amplifying message from a single cell, unequivocally demonstrates that our cultures did not contain endothelial cells. If these were contaminants in our eniched cell populations, even at a low level, they would have been detected by RT-PCR for factor VIII, particularly after 35 amplification cycles.

The finding of both VEGF protein and receptors within the same cell population suggests important autocrine/paracrine mechanisms which may be important in direct tumour cell proliferation. However, no proliferative response could be elicited when rhVEGF was added directly to primary cultures over a 4-day period. There are several possible explanations for this. Given that these cultures are already producing reasonably high levels of VEGF (as demonstrated by our ELISA results), it may be that the receptors are already maximally stimulated and have become saturated such that they would be unresponsive to exogeneous peptide. We did not have access to a VEGF antagonist which may have tested this hypothesis. Alternatively, VEGF receptors observed on breast tumour cells may be involved with invasion rather than proliferation, suggested in ovarian carcinoma which also possesses gene transcripts for both types of VEGF receptor, but cannot respond to exogenous rhVEGF in either paracrine or autocrine fashion (Boocock et al, 1995). It is also possible that the receptor gene may not be transcribed and translated to a functional state under the in vitro conditions we describe; expression of a functional receptor may require cell–cell or cell–matrix interactions (and hence tissue integrity), which is obviously lost in vitro. The soluble Flt-1 receptor (sFlt-1), identified in human endothelial cells, which binds VEGF with high affinity thus preventing VEGF-induced mitogenesis (Kendall et al, 1996) should also be considered. One could speculate that sFlt-1 may also be produced

© Cancer Research Campaign 1999

by breast primary cultures and, by binding to its ligand, may be responsible for the lack of proliferative effect we observed when exogenous VEGF was added to our cultures. However, a preliminary study (by sandwich ELISA) in our laboratory has failed to demonstrate the presence of sFlt-1 in samples of conditioned medium, although it is present in sera of some breast cancer patients (K Heer et al, unpublished observations).

A differential expresison of Flt-1 and KDR was observed using semi-quantitative RT-PCR, which may reflect a differential response to VEGF by specific cell types. We observed up-regulation of Flt-1 by breast stromal cells and this observation reflects recent data from an immunostaining study where there was a trend for Flt-1 to be associated with breast stroma rather that epithelial cells (51% and 44% expression respectively; de Jong et al, 1998). With KDR, the reverse was true, with very strong expression by epithelial cells. This observation has precedent, with the differential expression of VEGF receptors having been described in normal human testes where a paracrine mitogenic and angiogenic role has been proposed (Ergun et al, 1997). Flt-1 has a higher affinity for VEGF than KDR and each have different signalling pathways (Waltenberger et al, 1994; Seetharam et al, 1995). It is proposed that both are required to mediate the full spectrum of VEGF binding, but the mechanisms which control VEGF signal transduction are not fully understood.

Breast tumours express a wide range of cytokines, which are also expressed by breast primary cultures (Speirs et al, 1996*b*; Green et al, 1997). Some cytokines, in particular IL-6 and IL-8 are up-regulated in breast tumours (Green et al, 1997). As both of these factors are pro-angiogenic (Koch et al, 1992; Cohen et al, 1996), we examined the effect of IL-6 and IL-8 on VEGF production by primary cultures. In breast tumour epithelial-enriched cultures, neither IL-6 nor IL-8 affected VEGF production. This is in contrast to the work of Cohen (1996), who demonstrated that in tumour cell lines, expression of VEGF was induced by IL-6, particularly in responses to hypoxia. However, none of these cell lines were derived from breast tumours, so it is possible that cells of different tissue origin may elicit distinct response to cytokines. Alternatively, the response of established cell lines may differ from that of the primary cultures used in the present study. In stromal cultures VEGF production was reduced by up to 50% in response to these cytokines. Down-regulation of VEGF by IL-6 and IL-8 suggests that stromal cells may be important paracrine regulators of VEGF. In so doing they may reduce the availability of VEGF to the surrounding neovasculature and/or epithelial cells, possibly acting as mediators which inhibit tumour growth.

We attempted to correlate our data with available clinicopathological details. With respect to VEGF receptors, epithelial and stromal cultures from the single lobular carcinoma available for study failed to express Flt-1. This observation has previously been reported and may be explained by the fact that, unlike ductal carcinoma, in general lobular carcinoma does not induce a desmoplastic stromal response (Brown et al, 1995). In terms of VEGF production, no significant difference was observed between cultures derived from node-positive versus node-negative tumours. However, stromal cultures derived from oestrogen receptor-negative tumours produced more than double the amount of VEGF produced from identical cultures derived from oestrogen receptor-positive tumours. The significance of this observation remains to be determined.

The findings reported here are in accord with the established clinical importance of VEGF as an independent prognostic factor in breast cancer (Toi et al, 1993; Linderholm et al, 1998) and consistent with the significantly higher levels in the sera of breast cancer patients (Dirix et al, 1997). The data presented here have reinforced the importance of VEGF in breast cancer using an in vitro model. More importantly, we have demonstrated non-endothelial expression of VEGF receptors Flt-1 and KDR by breast cancer epithelial and stromal cells. Furthermore, production of VEGF by breast cancer stromal cells supplements a recent study where in transgenic mice bearing oncogene-induced mammary carcinomas, the VEGF promoter was expressed more strongly in tumour stroma (Fukumara et al, 1998), pointing to an important role for the stroma in VEGF production. In conclusion, these results suggest a fundamental role for VEGF in breast cancer, not only as an angiogenic factor, but also as an autocrine/paracrine regulator which may facilitate tumour proliferation and subsequent invasion.

### ACKNOWLEDGEMENTS

The authors wish to thank Mr PJ Carleton, Hull Royal Infirmary, for kindly providing us with breast tumour samples. This study was supported by Royal Hull Hospitals NHS Trust.

### REFERENCES

Adams EF, Rafferty B and White MC (1991) Interleukin-6 is secreted by breast fibroblasts and stimulates 17β-estradiol oxidoreductase activity of MCF-7 cells: possible paracrine regulation of breast 17β-estradiol levels. *Int J Cancer* **49**: 118–121

Boocock CA, Charnock-Jones S, Sharkey AM, McLaren J, Barker PJ, Wright KA, Twentyman PR and Smith SK (1995) Expression of vascular endothelial growth factor and its receptors flt and KDR in ovarian carcinoma. *J Natl Cancer Inst* **87**: 506–516

Brown LF, Berse B, Jackman RW, Tognazzi K, Guidi AJ, Dvorak HF, Senger DR, Connolly JL and Schnitt SJ (1995) Expression of vascular permeability factor (vascular endothelial growth factor) and its receptors in breast cancer. *Hum Pathol* **26**: 86–91

Charnock-Jones DS, Sharkey AM, Boocock CA, Ahmed A, Plevin R, Ferrara N and Smith SK (1994) Vascular endothelial growth factor receptor localisation and activation in human trophoblast and choriocarcinoma cells. *Biol Reprod* **51**: 524–530

Cohen T, Nahari D, Cerem LW, Neufeld G and Levi B-Z (1996) Interleukin-6 induces the expression of vascular endothelial growth factor. *J Biol Chem* **271**: 736–741

Dirix LY, Vermeulen PB, Pawinski A, Prove A, Benoy I, de Pooter C, Martin M and van Oosterom AT (1997) Elevated levels of the angiogenic cytokines basic fibroblast growth factor and vascular endothelial growth factor in sera of cancer patients. *Br J Cancer* **76**: 238–243

de Jong JS, van Diest PJ, van der Valk P and Baak JPA (1998) Expression of growth factors, growth inhibiting factors and their receptors in invasive breast cancer. I: An inventory in search of autocrine and paracrine loops. *J Pathol* **184**: 44–52

de Vries C, Escobedo JA, Ueno H, Houck K, Ferrara F and Williams LT (1992) The fms-like tyrosine kinase, a receptor for vascular endothelial growth factor. *Science* **255**: 989–991

Eppenberger U, Kueng W, Schlaeppi JM, Roesel JL, Benz C, Mueller H, Matter A, Zuber M, Luescher K, Litschgi M, Schmitt M, Foekens JA and Eppenberger-Castori S (1998) Markers of tumor angiogenesis and proteolysis independently define high- and low-risk subsets of node-negative breast cancer patients. *J Clin Oncol* **16**: 3129–3136

Ergun S, Kilic N, Fiedler W and Mukhopadhyay AK (1997) Vascular endothelial growth factor and its receptors in normal human testicular tissue. *Mol Cell Endocrinol* **131**: 9–20

Ferrara N and Davis-Smyth T (1997) The biology of vascular endothelial growth factor. *Endo Rev* **18**: 4–25

Folkman J (1990) What is the evidence that tumors are angiogenesis dependent? *J Natl Cancer Inst* **82**: 4–6

Fukamara D, Xavier R, Sugiura T, Chen Y, Park E-C, Lu N, Selig M, Nielsen G, Taksir T, Jain RK and Seed B (1998) Tumor induction of VEGF promoter activity in stromal cells. *Cell* **94**: 715–725

 © Cancer Research Campaign 1999

Gitay-Goren H, Halaban R and Neufeld G (1993) Human melanoma cells but not normal melanocytes express vascular endothelial growth factor receptors. *Biochem Biophys Res Commun* **190**: 702–708

Green AR, Green VL, White MC and Speirs V (1997) Expression of cytokine messenger RNA in normal and neoplastic human breast tissue: identification of interleukin-8 as a potential regulatory factor in breast tumours. *Int J Cancer* **72**: 937–941

Green VL, Atkin SL, Speirs V, Jeffreys RV, Landolt AM, Mathew B, Hipkin L and White MC (1996) Cytokine expression in human anterior pituitary adenomas. *Clin Endo* **45**: 179–185

Hewett PW and Murray JC (1996) Coexpression of flt-1, flt-4 and KDR in freshly isolated and cultured human endothelial cells. *Biochem Biophys Res Commun* **221**: 697–702

Hlatky L, Tsionou C, Hahnfeldt P and Coleman CN (1994) Mammary fibroblasts may influence breast tumour angiogenesis via hypoxia-induced vascular endothelial growth factor up-regulation and protein expression. *Cancer Res* **54**: 6083–6086

Jakeman LB, Winer J, Bennett GL, Altar CA and Ferrara N (1992) Binding sites for vascular endothelial growth factor are localised on endothelial cells in adult rat tissues. *J Clin Invest* **89**: 244–253

Kendall RL, Wang G and Thomas KA (1996) Identification of a soluble form of the vascular endothelial growth factor receptor flt-1 and its heterodimerisation with KDR. *Biochem Biophys Res Commun* **226**: 234–328

Klagsbrun M and Soker S (1993) VEGF/VPS: the angiogenesis factor found? *Curr Biol* **3**: 699–702

Koch AE, Polverini PJ, Kunkel SL, Harlow LA, DiPietro LA, Elner VM, Elner SG and Strieter RM (1992) Interleukin-8 as a macrophage-derived mediator of angiogenesis. *Science* **258**: 1798–1801

Linderholm B, Tavelin B, Grankvist K and Henriksson R (1998) Vascular endothelial growth factor is of high prognostic value in node-negative breast carcinoma. *J Clin Oncol* **16**: 3121–3128

Lu Q and Brodie A (1996) Stimulation of the growth of MCF-7 and MDA-MB-468 breast cancer cells by vascular endothelial growth factor. *Proc Am Assoc Cancer Res* **37**: 1499

Scott PAE, Smith K, Poulsom R, De Benedetti A, Bicknell R and Harris AL (1998) Differential expression of vascular endothelial growth factor mRNA vs. protein isoform expression in human breast cancer and relationship to elF-4E. *Br J Cancer* **77**: 2120–2128

Seetharam L, Gotoh N, Maru Y, Neufeld G, Yamaguchi S and Shibuya MA (1995) A unique signal transduction pathway for the flt-1 tyrosine kinase receptor, a receptor for vascular endothelial growth factor. *Oncogene* **10**: 135–147

Speirs V, Green AR and White MC (1996*a*) Collagenase III: a superior enzyme for complete disaggregation and improved viability of normal and malignant human breast tissue. *In Vitro Cell Dev Biol* **32**: 72–74

Speirs V, Green AR and White MC (1996*b*) A comparative study of cytokine gene transcripts in normal and malignant breast tissue and primary cell cultures derived from the same tissue samples. *Int J Cancer* **66**: 551–556

Speirs V, Green AR, Walton DS, Kerin MJ, Carleton PJ, Fox JN, Desai SB and Atkin SL (1998) Short-term primary culture of epithelial cells derived from breast tumours. *Br J Cancer* **78**: 1412–1429

Terman BI, Dougher-Vermazen M, Carrion ME, Dimitrov D, Armellino DC, Gospodarowicz D and Bohlen P (1992) Identification of the KDR tyrosine kinase as a receptor for vascular endothelial growth factor. *Biochem Biophys Res Commun* **187**: 1579–1586

Tischer E, Mitchell R, Hartman T, Silva M, Gospodarowicz D, Fides JC and Abraham JA (1991) The human gene for vascular endothelial growth factor. Multiple protein forms are encoded through alternative exon splicing. *J Biol Chem* **266**: 11947–11954

Toi M, Kashitani L and Tominaga T (1993) Tumour angiogenesis is an independent prognostic indicator in primary breast carcinoma. *Int J Cancer* **55**: 371–374

Toi M, Kondo S, Suzuki H, Yamamoto Y, Inada K, Imazawa T, Taniguchi T and Tominaga T (1996) Quantitative analysis of vascular endothelial growth factor in primary breast cancer. *Cancer* **77**: 1101–1106

Waltenberger J, Claesson-Welch L, Siegbahn A, Shibuya M and Heldin C-H (1994) Different signal transduction properties of KDR and Flt-1, two receptors for vascular endothelial growth factor. *J Biol Chem* **269**: 26988–26955

Yoshiji H, Gomez DE, Shibuya M and Thorgeirsson UP (1996) Expression of vascular endothelial growth factor, its receptor, and other angiogenic factors in human breast cancer. *Cancer Res* **56**: 2013–2016

Weidner N, Semple JP, Welch WR and Folkman J (1991) Tumour angiogenesis and metastasis: correlation in invasive breast cancer. *N Engl J Med* **324**: 1–8

*© Cancer Research Campaign 1999* 
